# Dyslipidaemia and Medical Outcome (Health Related Quality of Life) in Patients with Schizophrenia Taking Antipsychotics in Enugu, Nigeria

**DOI:** 10.1155/2017/9410575

**Published:** 2017-02-20

**Authors:** Emmanuel Omamurhomu Olose, John Edet, Monday Nwite Igwe, Donald Chidozie Chukwujekwu, Miriam Chinyere Aguocha, Richard Uwakwe

**Affiliations:** ^1^Department of Psychiatry, University of Calabar, Calabar, Nigeria; ^2^Department of Clinical Services, Federal Neuropsychiatric Hospital, Calabar, Nigeria; ^3^Department of Psychiatry, Ebonyi State University, Abakaliki, Nigeria; ^4^Department of Psychiatry, University of Port Harcourt, Port Harcourt, Nigeria; ^5^Department of Psychiatry, Imo State University, Orlu, Nigeria; ^6^Department of Psychiatry, Nnamdi Azikiwe University, Awka, Nigeria

## Abstract

*Aim*. Determine association between use (and type) of antipsychotics and dyslipidaemia in newly diagnosed schizophrenia patients attending Federal Neuropsychiatric Hospital, Enugu.* Methods*. From sixty antipsychotic naive patients with schizophrenia and sixty first-degree relatives matched for gender and age, fasting blood lipid profiles were measured at baseline and after twelve weeks. Medical Outcome Study Short Form General Health Survey was administered to patients on both occasions. Fasting lipid profile changes of both groups were compared.* Results*. Mean endpoint of total cholesterol (TC), low density lipoprotein (LD), and triglycerides (TG) in mmol/l for cases was significantly higher than initial values (TC 4.5 versus 4.3, *t* = 4.3, *p* < 0.0001), (LDL 2.8 versus 2.6, *t* = 14.3, *p* < 0.0001), and (TG 1.3 versus 1.0, *t* = 12.1, *p* < 0.0001). Mean endpoint of high density lipoprotein (HDL) in mmol/l for cases was significantly lower than initial values (1.1 versus 1.2, *t* = 12.1, *p* < 0.0001). Prevalence of dyslipidaemia for cases was 13%. Mean endpoint of TC, LDL, TG, and HDL in mmol/l for controls was not significantly different from initial values (TC 4.30 versus 4.27, *t* = 1.09, *p* = 0.279), (LDL 2.49 versus 2.46, *t* = 1.28, *p* = 0.205), (TG 0.96 versus 0.94, *t* = 1.27, *p* = 0.207), and (HDL 1.37 versus 1.38, *t* = 1.61, *p* = 0.113). Subjects on atypical antipsychotics had higher risk for dyslipidaemia.* Conclusion*. Use of antipsychotics was significantly associated with dyslipidaemia.

## 1. Introduction

Dyslipidaemia and lipid metabolism have attracted attention of researchers in recent times. For patients with mental disorders, the observation that the disease itself and its treatment may be associated with metabolic problems adds another layer of complexity. Abnormal lipid levels have been unequivocally associated with increased risk for cardiovascular events such as myocardial infarction and stroke in the general population [1]. They also contribute additional risk factors to patients with diabetes mellitus and coronary heart disease [2]. Patients with schizophrenia have a life expectancy 20% shorter than that of the general population [3]; approximately 2/3 of the excess mortality is due to comorbid medical illness [4]. The rate of cardiovascular mortality in patients with schizophrenia is twice that seen in the general population. Antipsychotic treatment is associated with various degrees of weight gain and dyslipidaemia [5], which may possibly contribute to excess mortality in patients with schizophrenia.

Dyslipidaemia was defined as an abnormality of any of the lipid profile components, that is, elevated total cholesterol (TC) ≥ 5.2 mmol/l, low density lipoprotein cholesterol ≤ 3.4 mmol/l, triglycerides (TG) ≥ 1.7 mmol/l, and high density lipoprotein (HDL) < 1.04 mmol/l.

Patients with schizophrenia are at an increased risk of dyslipidaemia in part due to poor diet and sedentary life style [6], antipsychotic treatment is also associated with various degrees of dyslipidaemia. Elevated blood lipids particularly triglycerides are associated with some typical antipsychotics, shortly after their introduction phenothiazines were found to elevate serum triglycerides and total cholesterol levels [7]. Clozapine and olanzapine have been shown to cause significant hypertriglyceridaemia compared with typical antipsychotics. The atypicals vary in their propensity to cause weight gain; the difference in weight gain associated with these agents reflects their order of risk for insulin resistance and dyslipidaemia [8].

Given that the majority of patients with schizophrenia face chronic, even life-long treatment with APDs, the risks of metabolic symptom such as dyslipidaemia are major considerations for individuals on APD maintenance treatment.

In Nigeria studies on antipsychotic associated dyslipidaemia are scanty and the few studies are mainly cross sectional. There is a need for more longitudinal prospective studies. The present study was designed to determine the prevalence of dyslipidaemia as well as association between the use of antipsychotic drugs and dyslipidaemia in newly diagnosed patients with schizophrenia.

## 2. Aims and Objectives

*The general aim* of the study was to determine the relationship between the use of antipsychotic drugs and dyslipidaemia in newly diagnosed patients with schizophrenia attending the Federal Neuropsychiatric Hospital, Enugu.

The specific objectives of the study were the following:

1. To determine the association between use (and type) of antipsychotic and dyslipidaemia in newly diagnosed patients with schizophrenia attending the Federal Neuropsychiatric Hospital, Enugu.
2. To compare the patients' fasting lipid profile with those of first-degree relations not using antipsychotic medication.
3. To determine the prevalence of dyslipidaemia in patients with schizophrenia using antipsychotic medications.
4. To determine the medical outcome (health related quality of life both mental and physical) in patients with schizophrenia attending the Federal Neuropsychiatric Hospital, Enugu.

## 3. Methodology

### 3.1. Setting of the Study

This was a prospective study carried out at the Federal Neuropsychiatric Hospital, Enugu. The Federal Neuropsychiatric Hospital, Enugu, is the only Federal government owned psychiatric hospital in the south east geopolitical zone of Nigeria. The hospital also receives patients from Delta, Rivers, Kogi, and Benue states. On the average the hospital attends to about one thousand five hundred patients monthly out of whom about 320 are new patients.

### 3.2. Ethical Issues

All patients and their next of kin gave signed informed consent to participate in the study which commenced after approval was obtained from the ethics committee of the Federal Neuropsychiatric Hospital, Enugu. Ethical clearance was obtained on the 19th day of December 2013. Refusal of a patient to participate or continue in the study at any point did not affect the quality of care received from the hospital. Those found to have lipid profiles of clinical significance were discussed with the consultants/doctors in charge with a view to having the patients referred for further assessment and management. The researchers bore the cost for payment of lipid profiles analysis.

### 3.3. Sample Size

A minimum required sample size was calculated using the computer software (Java Stat) for comparative control study. This gave a minimum sample size of 57 for each group of participants to be studied. For the purpose of this study the sample size was inflated by 20% for each group to make more robust the findings that would be generated. Thus a total of 136 participants were recruited for the study consisting of 68 patients with schizophrenia and 68 first-degree relations (controls) to eliminate genetics as a confounding factor.

### 3.4. Eligibility Criteria

#### 3.4.1. Inclusion Criteria [Patients]

1. Participants meeting ICD-10 diagnostic criteria for schizophrenia attending hospital for the first time.
2. Participants who are antipsychotic naive.
3. Age, ≥ 18 years.
4. Must have a first-degree relation ready to take part in the project.
5. Must give informed consent.

#### 3.4.2. Inclusion Criteria [Controls]

1. First-degree relations [siblings] matched for gender and age ±5 years.
2. Age ≥ 18 years.
3. Must give informed consent.

#### 3.4.3. Exclusion Criteria [Patients and Controls]

1. Medical conditions related to dyslipidemia (e.g., obesity, i.e., body mass index [BMI] greater than 30 at entry point, diabetes mellitus) as well as abnormal lipid profile at the point of entry.
2. Taking medications related to dyslipidemia (e.g., oral contraceptives, thiazides, and steroids).
3. Those who refuse to give consent.

### 3.5. Instruments

#### 3.5.1. Sociodemographic Interview Schedule

This was designed by the author to obtain basic information about the participant's health and biodata. General medical diagnosis, medication in use including duration of use as well as any illness common in the family was documented. The antipsychotics as well as the dosages prescribed were noted.

#### 3.5.2. M-CIDI (Munich Version of the Composite International Diagnostic Interview)

M-CIDI was produced for the World Health Organization (WHO) and the United States (US) Alcohol Drug Abuse and Mental Health Administration by the US National Institute of Mental Health. It is used for the assessment of mental disorders and to provide diagnoses according to ICD10 and DSM IV. The CIDI package comes in modules. In this study only the schizophrenia module was used. The M-CIDI has been used previously in this environment by Olose et al. [9].

#### 3.5.3. Medical Outcome Study Short Form General Health Survey (SF-12)

SF-12 was developed by Stewart et al. [10]. The SF-12 Health Survey is a 12-item subset of the SF-36 that measures the same eight domains of health. It is a brief, reliable measure of overall health status of the respondent. The instrument can either be self-administered or given by a trained interviewer in person or by telephone to persons aged 14 and older. It takes about 2 to 3 minutes to complete. The categories/domains of health which the instrument measures are: Physical functioning, Role physical, Bodily pain, General health, Vitality, Social functioning, Role-emotional and mental health. These domains are scaled on 2 and 5-point Likert scale. The SF-12 Health Survey is published in both standard (4-week) and acute (1-week) recall versions for self-administration as well as scripts for personal interviews. The SF-12 physical and mental health summary measures are referred to as PCS-12 and MCS-12, respectively. The standard 4-week recall period was adapted for the SF36 and SF-12 to maintain comparability with the long -form Medical outcome Study (MOS) measures from which it was derived. The test-retest reliability of the PCS-12 summary measures was 0.890 in the US and 0.864 in the UK. Coefficients of 0.760 in US and 0.774 in UK were observed for the MCS-12. Fourteen validity tests involving physical criteria ranged from 0.43 to 0.93 (median = 0.6 7).

The literature has shown that concurrent validity for both the short form 12 and the short form 20 is the same in psychometric properties. The SF-20 has been validated in Nigeria [11]. In the present study the SF-12 was used to determine the overall health related status/outcome of patients.

#### 3.5.4. Method of Lipid Profile Estimation

The method of Trinder [12] was used for the estimation of total cholesterol.

Triglyceride values were estimated by the method of Barham and Trinder [13]. LDL cholesterol was calculated by the Friedewald equation [14]: LDL = TC − TG/2.2 − HD.

#### 3.5.5. Participant Selection and Sampling Technique

*Patients*. All patients with schizophrenia coming to the emergency clinic were first of all identified. Those coming for the first time were then specifically identified to determine their eligibility for inclusion. Participants that met the inclusion criteria were then traced with the assistance of medical record staff to the various service points of the hospital that is the emergency ward, inpatient ward and the outpatient department. Consecutive attendees with schizophrenia presenting for the first time were approached for recruitment.

*Controls*. For all the selected participants, efforts were made to get a first-degree biological relative, to eliminate genetics as a confounding factor after matching for gender and age (±5 years).

### 3.6. Procedure

Data collection took place from December 2013 to June 2014. The first step was to identify patients diagnosed as having schizophrenia by the doctor on duty at the emergency room.

When the participants (patients) had been identified and selected explanation of the study and how he/she was selected were proffered. Informed consent was then obtained; this was followed by administration of M-CIDI to confirm the diagnosis of schizophrenia in the patients. After patients were identified, a first-degree relative (matched for gender and age ±5 years) for whom no diagnosis of schizophrenia had been made from history and who took no antipsychotic agent was identified if they met the inclusion criteria. Sociodemographic data of age, educational status, occupation, and family history of cardiovascular disease or diabetes mellitus was then obtained. The Medical Outcome Study Short Form General Health Survey (SF-12) was also administered to all the patients. A single venous blood sample was obtained for fasting lipid profile analysis. All blood samples were processed by the same Federal Neuropsychiatric Hospital laboratory Scientist. The same procedure was carried out for the controls. At the end of 12 weeks, all the outcome measures were repeated for the patients and the control groups. At the second assessment one hundred and twenty subjects remained in the study that is sixty cases and sixty controls. Sixteen subjects dropped out of the study. Patients were categorized according to the antipsychotics (typical or atypical) that they used in the previous 12 weeks before the second assessment. As a naturalistic study the attending doctor determined the prescription of antipsychotics. Any drugs used in the preceding twelve weeks were recorded.

### 3.7. Statistical Analysis

Data was analyzed using the personal computer version of the Statistical Package for the Social Sciences (SPSS- PC version 20). The subjects were classified according to their lipid profile status, use of antipsychotics, and sociodemographic variables. All the antipsychotics used by the index cases were converted to their CPZ equivalent doses. Noncontinuous variables were compared using Chi-square test while the *t*-test was used for continuous variables such as age and lipid levels.

For the individual antipsychotics the mean baseline fasting blood lipids values were compared with the respective mean endpoint values by using *t*-test. All tests were two-tailed with significance level set at 0.05%.

## 4. Results

### 4.1. Sociodemographic Profile of Study (Patients) and Controls

See Table 1.

#### 4.1.1. Antipsychotic Usage and Dosage

The cases were prescribed either typical or atypical antipsychotic drugs. Among the cases who used typical antipsychotic drugs, 39 (83%) were prescribed haloperidol, 5 (10.6%) were prescribed chlorpromazine while only 3 (6.4%) cases took trifluoperazine.

Among the 13 (21.6%) cases placed on atypical antipsychotics 9 (69%) were prescribed olanzapine and 4 (31%) took risperidone.

The mean dosage per day in milligram for cases using typical antipsychotics was 340 mg/day for those prescribed chlorpromazine and 18.46 mg/day for those prescribed haloperidol while those on trifluoperazine were prescribed 16.67 mg/day. The mean chlorpromazine equivalent dosage was 615.33 and 333.40 for those on haloperidol and trifluoperazine, respectively.

For cases prescribed atypical antipsychotics the mean daily dosage was 18.33 mg/day with a range of 15 to 20 mg for cases placed on olanzapine, six cases took 20 mg olanzapine daily while three took 15 mg of olanzapine daily. While those on risperidone received a mean daily dosage of 3.75 mg/day with a range of 3 to 4 mg, 3 cases received 4 mg/day and one received 3 mg/day. The chlorpromazine equivalent dosage for patients on olanzapine and risperidone was 366.6 and 375, respectively.

#### 4.1.2. Association between the Use of Antipsychotics and Dyslipidaemia

The mean endpoint of total cholesterol (TC) for cases was significantly higher than the baseline (4.50 mmol/l versus 4.34 mmol/l, = 4.317, *p* < 0.001).

The mean endpoint of total cholesterol (TC) for controls was not significantly different from baseline (4.27 mmol/l versus 4.30 mmol/l, = 1.093, *p* = 0.279).

The mean endpoint of triglyceride (TG) for cases was significantly higher than the baseline (1.28 mmol/l versus 1.05 mmol/l, = 4.371, *p* < 0.0001).

The mean endpoint of TG for controls was not significantly different from baseline (0.96 mmol/l versus 0.94, = 1.276, *p* = 0.207).

The mean endpoint of high density lipoproteins (HDL) for cases was significantly lower than baseline (1.11 versus 1.23, = 12.164, *p* < 0.0001).

The mean endpoint of HDL for controls was not significantly different from baseline (1.37 versus 1.38, = 1.61, *p* = 0.113).

The mean endpoint of low density lipoprotein (LDL) for cases was significantly higher than baseline (2.78 versus 2.64, = 4.000, *p* < 0.0001).

The mean endpoint of LDL for controls was not significantly different from baseline (2.49 mmol/l versus 2.46 mmol/l, = 1.280, *p* = 0.205).

Eight (13.3%) of the cases (*n* = 60) developed dyslipidaemia whereas the lipid profiles of the controls remained within normal limits.

#### 4.1.3. Association between the Class or Type of Antipsychotic and Dyslipidaemia

Among the thirteen (21.66%) cases that received atypical antipsychotics, 3 (23.08%) developed dyslipidaemia. From the 47 (78.34%) that received typical antipsychotics 5 (10.64%) developed dyslipidaemia (*x*^2^ = 1, *p* value = 0.356).

The relative risk (risk ratio) for the development of dyslipidaemia in atypical versus typical antipsychotics was 1.16 (95% CI = 0.85–1.60) There was an increased risk of dyslipidaemia with atypical antipsychotics compared to typical antipsychotic therapy OR = 2.52, 95% CI = 0.51 to 12.35.

#### 4.1.4. Individual Antipsychotics and Change in Lipid Profile

Among the cases that received typical antipsychotics 2 (40%) on chlorpromazine (*n* = 5) developed dyslipidaemia. Chlorpromazine's main effect was on the triglyceride fraction which was markedly raised (mean change = 0.34 mmol/l) and the HDL fraction which was markedly lowered (mean change = 0.30 mmol/l). The mean change in TC and LDL was an increase of 0.05 mmol/l and 0.14 mmol/l, respectively (Table 2).

Three (7.69%) cases on haloperidol (*n* = 39) developed dyslipidaemia. The mean increase for TC, TG, and LDL was 0.09 mmol/l, 0.18 mmol/l, and 0.09 mmol/l, respectively; the mean decrease for HDL was 0.08 mmol/l.

None of the cases on trifluoperazine (*n* = 3) developed dyslipidaemia. There was no mean change in the LDL fraction. The mean increase for TC and TG was 0.02 mmol/l and 0.23 mmol/l, respectively. The mean decrease in HDL was 0.13 mmol/l (Table 2). Among the patients on atypical antipsychotics olanzapine caused the most significant increase in serum TC, LDL, and TG (0.56 mmol/l, 0.40 mmol/l, and 0.40 mmol/l, resp.) and decrease in HDL (0.18 mmol/l) (Table 2).

Three (33.33%) patients on olanzapine (*n* = 9) developed dyslipidaemia. None of the patients on risperidone (*n* = 4) developed dyslipidaemia, and they had a mean increase in serum TC, LDL, and TG of 0.16 mmol/l, 0.14 mmol/l, and 0.23 mmol/l, respectively. The mean decrease in HDL was 0.12 mmol/l (Table 2).

#### 4.1.5. Short Form 12 Medical Outcome of Patients

The Short Form 12 PCS baseline mean value for patients was 52.84 (SD = 5.54) with a range of 31.70 to 59.20. The endpoint PCS12 mean value was 52.07 (SD = 5.39) with a range of 34.20 to 58.0. There was no significant difference between the mean baseline and endpoint PCS of cases (*t* = .858  *p* = 0.394). The mean baseline short form 12 MCS components for cases was 34.60 (SD = 9.753) with a range of 13 to 55. The mean endpoint short form 12 MCS component was 51.50 SD = 4.70 with a range of 37.30 to 58.50. There was a significant difference between the mean baseline and endpoint MCS (*t* = 168, df = 59, *p* = 0.0001).

For patients placed on typical antipsychotics, the mean baseline SF 12 PCS component was 52.67 (SD = 6.02) with a range of 31.70 to 59.20. The mean endpoint PCS component was 51.58 (SD = 5.50) with a range of 34.20 to 58.0. There was no significant difference between the baseline and endpoint PCS scores (*t* = 0.768, *p* = 0.446). For typical antipsychotics, the mean baseline short form 12 MCS component was 34.72 (SD = 10.59) with a range of 34.20 to 58. The endpoint mean SF 12 MCS was 51.55 (SD = 4.73) with a range of 37.30 to 58.10. Endpoint MCS score was significantly higher than baseline (*t* = 12.942, df = 46, *p* value < 0.0001).

For patients placed on atypical antipsychotics the mean baseline SF-12 PCS component was 53.43 SD = 3.40, range 47.20 to 59.20. The mean endpoint PCS component was 53.0 SD = 4.61 range 42.60 to 57.90. There was no significant difference between baseline and endpoint PCS scores (*t* = 0.508, *p* = 0.621). The mean baseline MCS was 34.18 SD = 6.20 with a range of 26.80 to 44.70. The mean endpoint MCS was 51.32 SD = 4.96 with a range of 41.20 to 58.50. Endpoint MCS score was significantly higher compared to baseline (*t* = 7.892, *p* < 0.0001).

## 5. Discussion

### 5.1. Association between the Use of Antipsychotics and Dyslipidaemia

The present study found significant changes in lipid profile (an increase in TC, TG, LDL and decrease in HDL levels) at the end of 12 weeks among cases but no significant change among the controls. This finding is similar to that of an American study by Wirshing et al. [15] which compared the effects of atypical antipsychotics clozapine, risperidone, quetiapine, and olanzapine and typical antipsychotics haloperidol and fluphenazine on glucose and lipid profile. Two hundred and fifteen patients were reviewed from two and half years before and after initiation of antipsychotic treatment. Covariates including patients other medications were controlled for in the analysis. All the groups had significant increases in lipid profiles.

The findings in the present study are also similar to the findings in the Iranian study carried out at the cardiovascular research Centre Isfahan University of Medical Sciences by Hamidreeza et al. [16]. The objective of the Iranian study was to compare the effects of atypical and typical antipsychotics on lipid profiles. One hundred and twenty-eight patients with schizophrenia were enrolled in the study and were divided into two groups. There was no significant difference in age, gender, duration of illness, period of drug consumption and age at onset of illness in the two groups. It was found that the level of lipid profile increased in both typical and atypical users.

The findings in the present study are similar to that of a Nigerian study [17] which compared the effects of clozapine and risperidone on lipid profiles. At the end of six weeks there was a significant increase in serum TC, LDL, TG and a significant decrease in HDL in both treatment groups compared to controls.

Meyer and Koro [18] reviewed the literature since 1970 documenting the effects of antipsychotic agents on serum lipids; both typical and atypical antipsychotics were associated with lipid dysregulation. This finding is in line with the present study.

However, Smith et al. [19] conducted a comparative study of atypical antipsychotics including clozapine, risperidone, and olanzapine and typical antipsychotics and reported that most mean fasting lipid levels were within normal range and were not significantly different across the four treatment groups.

This difference in results obtained in the Smith study compared to the present study may be accounted for by the fact that in the present study only antipsychotics naive patients were enrolled, whereas in the study by Smith et al., patients with chronic schizophrenia who have been on long term therapy with antipsychotics were recruited. It has been shown that patients with schizophrenia treated for years with antipsychotic medication may develop tolerance to the effects of antipsychotics on lipid levels, so measurements of postprandial levels in addition to fasting levels may be helpful in identifying metabolic effects among such patients [17].

In the present study, 8 (13%) cases developed an abnormal lipid profile. This level is much higher than that obtained by Meyer and Koro [18] which was 7%. This difference may be accounted for by the fact that, in the Meyer and Koro study cases of dyslipidaemia were mainly defined as those who had received treatment for dyslipidaemia which may have omitted those with dyslipidaemia that did not receive treatment. In addition the study was also retrospective in nature. It has been shown that retrospective studies lack accuracy when compared to prospective studies [20].

In a study by Meyer and Koro [18], a prevalence of dyslipidemia of 23% was reported. The higher prevalence of dyslipidaemia compared to the present study may be explained by the fact that in that study mainly atypical antipsychotics were used whereas in the present study only 18% of the cases were placed on atypicals and the majority of the patients were on haloperidol. Meyer and Koro [18] in a review of the literature from 1970 to 2004 found that high potency conventional antipsychotics (e.g., haloperidol) appear to be associated with lower risk of dyslipidaemia compared to atypicals such as clozapine and olanzapine.

In a study by Lawani et al. [21] on the prevalence of metabolic syndrome in schizophrenic patients on antipsychotics in a Nigerian hospital a prevalence of 56% dyslipidaemia was obtained. The high level of dyslipidaemia seen in that study compared to the present study may be due to the duration of treatment which was 1–39 years with a mean duration of 6.09 years. The duration of use of antipsychotics is a determinant for the development of lipid abnormalities among patients with schizophrenia [18]. Also in the present study patients were screened for dyslipidaemia and some of its associated risk factors; patients with dyslipidaemia were omitted at baseline, whereas the Lawani et al. study was a cross sectional study without screening for metabolic risk factors.

The exclusion of patients with dyslipidaemia and obesity at baseline (onset of study) ensures that any dyslipidaemia noticed at the end of the study period is a likely reflection of the effect of the prescribed antipsychotic medication on the patients lipid profile. Although not sufficient to establish causality, this eliminates the likelihood for the occurrence of a type II error in this study. This is one of the strengths of this study unlike the situation in the study by Lawani et al. [21] mentioned above.

Different studies give different prevalence levels of dyslipidaemia and this probably may be accounted for by the different definitions of dyslipidemia in the studies as well as differences in ethnicity, sample size, drug used in the various studies, and duration of follow-up.

### 5.2. Association between the Class of or Type of Antipsychotic and Dyslipidemia

Although, in the present study, a greater percentage of cases receiving atypical antipsychotics (23.07%) developed dyslipidemia in comparison to 10.64% of those receiving typical antipsychotics, the association is not statistically significant. It seems that the effect of antipsychotics on lipid profiles depends on the individual antipsychotics and is not a class effect. The lack of class effect is highly consistent with the Joint Consensus Statement [22] issued by the American Diabetes Association, the American Psychiatric Association, the American Association of Clinical Endocrinologists and the North American Association for the study of obesity. This consensus statement issued in 2004 classified clozapine and olanzapine as being associated with an increased risk of worsening lipid profiles, whereas aripiprazole and ziprasidone were not associated with an increased risk for detrimental serum lipid changes. The statement noted that there were conflicting data about the effects of quetiapine and risperidone on lipids.

Furthermore, Lidenmeyer et al. [23] found significant increases in mean total cholesterol after treatment with olanzapine and clozapine but there was no elevation with haloperidol or risperidone, thus even though risperidone is an atypical antipsychotic it showed minimal effects on cholesterol levels.

In the present study, lipid profile increased in both typical and atypical antipsychotic users. This result is similar to a previous Iranian study by Hamidreeza et al. [16] which showed an increase in lipid profile in both the typical and atypical drug users. In that study the typical group had significantly higher mean TC and LDL levels than the atypical group, which could probably be explained by the fact that in the typical group 81.3% of the patients used phenothiazines, whereas in the present study in the typical group majority (82%) used haloperidol. Butyrophenones (Haloperidol) are associated with less chances of significant increase in visceral obesity which may be an important factor for development of insulin resistance and the metabolic syndrome [23]. These two conditions are associated with dyslipidemia which may probably explain the difference in study outcome of the Hamidreeza et al. study showing higher lipid profile levels in the typical antipsychotic group.

Among the atypical antipsychotics, olanzapine had a greater effect on lipid levels in the present study. The effects of olanzapine and risperidone exposure were evaluated in a large health care data base (the UK General Practice Research Database) [UK GPRD] [17]. Olanzapine was associated with a 3 fold risk in the odds of developing hyperlipidaemia compared with typical antipsychotics. Risperidone was not associated with increased odds of developing hyperlipidaemia compared with no antipsychotic or typical antipsychotic exposure. The UK GPRD result is consistent with the present study. In the present study the elevation of triglyceride was 14% with risperidone; other researchers have reported levels of 20% [24].

Meyer and Koro [18] in a retrospective study demonstrated that risperidone did not have any significant effect on serum cholesterol but caused concurrent increases in triglyceride levels. Meyer and Koro study also demonstrated a significant increase in levels of both triglycerides and cholesterol in olanzapine treated patients. These findings are in line with the present study.

Several other studies have confirmed the significant effect of olanzapine on the rise in serum lipids that is triglycerides [25], total cholesterol [25], LDL cholesterol [26], and HDL decline [27]. These are similar to the findings in the present study.

Among the typical antipsychotics used in the present study, chlorpromazine produced the greatest change in lipid profiles. There was a significant rise in total cholesterol and triglycerides as well as a significant reduction of HDL which has been demonstrated in previous studies [28, 29].

In the present study, haloperidol had the least effect on lipid profiles which is in line with the study by Krakowski et al. [30] that showed minimal change in all metabolic parameters with haloperidol after 3 months of treatment.

### 5.3. Medical Outcome Survey 12-Item Short Form Health Survey (SF-12) for Patients

The SF-12 scores have been normalised to yield a mean of 50 and a standard deviation of ten in the United States with higher scores indicating better functioning.

The physical component summary (PCS) baseline mean value of 52.84 (SD 5.54) did not differ significantly from the endpoint value 52.07 (SD 5.39). Thus, the effect of antipsychotics did not significantly affect the PCS component, though a few patients scored less after 12 weeks of medication probably due to side effects of medication whereas some improved.

The baseline mean (mental component summary) MCS component for patients (34.60 SD 9.75) differed significantly from the mean endpoint MCS component which was 51.50 (SD 4.54). Thus the endpoint MCS component for patients was significantly higher than baseline probably reflecting the effect of antipsychotics on symptom improvement leading to an improvement in the patient's health related quality of life.

The mean endpoint PCS for typical antipsychotic was 51.58; for atypical it was 52.8. The mean endpoint MCS for typical was 51.87, for atypical it was 51.42. Thus, it can be seen that the use of antipsychotics was associated with an improvement in the health related quality of life as measured by SF-12; it improved equally in those on both typical and atypical antipsychotics.

Patient's health related quality of life (HRQOL) may be affected directly by the treatments used to manage them. While antipsychotic medications have a positive effect due to symptom improvement, the side effect of antipsychotics such as weight gain and obesity would be expected to negatively affect functional status and overall quality of life. The deleterious side effects of antipsychotic medications, such as obesity negatively affecting functional status were not seen in this study probably because of the short duration. The final SF 12 values in the present study were much higher than in a study by George et al. [31]. The study by George et al. evaluated the health related quality of life (HRQOL) among patients treated with lurasidone. Stable but symptomatic out patients were switched from their current antipsychotics in a six week open label trial. Mental and physical component summary scores (MCS and PCS) were assessed using SF-12. Change in HRQOL from baseline to endpoint were compared using ANCOVA. Improvements in SF-12 MCS scores were observed (in the study by George et al.) for all patients and those switched from quetiapine or aripiprazole and the SF-12 PCS scores remained comparable to baseline in all patient groups. The higher SF 12 seen in the present study compared to the George et al. study may be due to the fact that George et al. used patients with a mean age of forty three who had been ill for some time and had probably developed negative symptoms. Additionally, the present study result may possibly reflect better prognosis of schizophrenia in developing countries [32].

## Figures and Tables

**Table 1:** Sociodemographic profile.

Variable	Frequency		Statistic	*p* value
	Study *N* = 60 (%)	Control *N* = 60 (%)		
*Gender*				
Male	27 (45)	27 (45%)	*x* ^2^ = 0.00	1.0
Female	33 (55)	33 (55%)		
*Age group *				
18–22	16 (26.7)	12 (19.9)	*t* = 0.063	0.950
23–27	19 (31.6)	23 (38.4)		
28–32	16 (26.7)	16 (26.7)		
33–37	6 (10)	6 (10.0)		
38–42	3 (5)	3 (5)		
Mean age	27.00 (5.38)	27.03 (5.80)		
*Marital status*				
Single	50 (83.3)	39 (65)	*x* ^2^ = 5.263	0.0218
Married	10 (16.7)	21 (35)		
*Education *				
Yes	59 (98.3)	60 (100)	*x* ^2^ = 1.008	0.3153
No	1 (1.7)	0		
*Occupation*				
Employed	8 (13.3)	32 (53.3)	*x* ^2^ = 27.19	0.0001
Unemployed	37 (61.7)	12 (20.0)		
Student	15 (25.0)	16 (26.7)		

**Table 2:** Individual antipsychotics and change in lipid profile.

Variable	Mean baseline (SD)	Mean end point (SD)	Mean change	*t*-test	df	*p*
*Haloperidol *						
TC (mmol/L)	4.35 (.42)	4.44 (.43)	0.09	2.172	39	0.036
TG (mmol/L)	1.07 (.25)	1.25 (.30)	0.18	13.314	39	0.0001
HDL (mmol/L)	1.23 (.12)	1.15 (.12)	0.08	15.632	39	0.0001
LDL (mmol/L)	2.63 (.37)	2.72 (.39)	0.09	2.182	39	0.035
*Chlorpromazine *						
TC (mmol/L)	4.30 (.45)	4.35 (.65)	0.05	0.405	4	0.706
TG (mmol/L)	1.11 (.26)	1.45 (.05)	0.34	4.687	4	0.009
HDL (mmol/L)	1.34 (.16)	1.04 (.00)	0.30	7.511	4	0.002
LDL (mmol/L)	2.43 (.37)	2.57 (.56)	0.14	1.057	4	0.350
*Trifluoperazine *						
TC (mmol/L)	4.14 (.67)	4.12 (.58)	0.02	.300	2	0.792
TG (mmol/L)	1.02 (.31)	1.25 (.39)	0.23	5.111	2	0.036
HDL (mmol/L)	1.20 (.05)	1.07 (.04)	0.13	4.298	2	0.650
LDL (mmol/L)	2.46 (.49)	2.46 (.04)	0.00	0.000	2	1.000
*Olanzapine *						
TC (mmol/L)	4.45 (.26)	5.01 (.26)	0.56	18.068	8	0.001
TG (mmol/L)	1.00 (.15)	1.40 (.19)	0.40	18.711	8	0.001
HDL (mmol/L)	1.22 (.04)	1.04 (.04)	0.18	17.097	8	0.001
LDL (mmol/L)	2.83 (.23)	3.23 (.39)	0.40	4.724	8	0.001
*Risperidone *						
TC (mmol/L)	4.29 (.39)	4.49 (.40)	0.20	11.286	3	0.001
TG (mmol/L)	.99 (.17)	1.14 (.22)	0.15	4.469	3	0.021
HDL (mmol/L)	1.16 (.05)	1.08 (.05)	0.08	0.200	3	0.008
LDL (mmol/L)	2.74 (.39)	2.87 (.30)	0.13	2.532	2	0.085

TC = total cholesterol, TG = triglycerides, HDL = high density lipoprotein, LDL = low density lipoproteins, change = change in lipid profile (mmol/L).

## Additional Points

*Limitations*. (1) The duration of follow-up was short. (2) This study did not control for important confounding factors such as diet, smoking, alcohol consumption, and levels of physical activity; therefore one cannot draw direct causal inferences. (3) This is a hospital based study and findings may not be generalized to the general population. In spite of these limitations, the findings from this study can serve as an important reference point for future epidemiological studies.* Strengths. *(1) Use of drug naive patients as prior use of antipsychotics may mask metabolic effects. (2) It used biological relations as comparison to eliminate genetics as a confounding factor. (3) It has the prospective longitudinal design. (4) This is a naturalistic study so the use and dosages of antipsychotics were determined by the attending doctors.
